# The HEART Mobile Phone Trial: The Partial Mediating Effects of Self-Efficacy on Physical Activity among Cardiac Patients

**DOI:** 10.3389/fpubh.2014.00056

**Published:** 2014-05-27

**Authors:** Ralph Maddison, Leila Pfaeffli, Ralph Stewart, Andrew Kerr, Yannan Jiang, Jonathan Rawstorn, Karen Carter, Robyn Whittaker

**Affiliations:** ^1^National Institute for Health Innovation, University of Auckland, Auckland, New Zealand; ^2^Auckland City Hospital, Auckland, New Zealand; ^3^Epidemiology and Biostatistics, University of Auckland, Auckland, New Zealand

**Keywords:** mobile phones, exercise, behavior, self-efficacy

## Abstract

**Background:** The ubiquitous use of mobile phones provides an ideal opportunity to deliver interventions to increase physical activity levels. Understanding potential mediators of such interventions is needed to increase their effectiveness. A recent randomized controlled trial of a mobile phone and Internet (mHealth) intervention was conducted in New Zealand to determine the effectiveness on exercise capacity and physical activity levels in addition to current cardiac rehabilitation (CR) services for people (*n* = 171) with ischemic heart disease. Significant intervention effect was observed for self-reported leisure-time physical activity and walking, but not peak oxygen uptake at 24 weeks. There was also significant improvement in self-efficacy.

**Objective:** To evaluate the mediating effect of self-efficacy on physical activity levels in an mHealth delivered exercise CR program.

**Methods:** Treatment evaluations were performed on the principle of intention to treat. Adjusted regression analyses were conducted to evaluate the main treatment effect on leisure-time physical activity and walking at 24 weeks, with and without change in self-efficacy as the mediator of interest.

**Results:** Change in self-efficacy at 24 weeks significantly mediated the treatment effect on leisure-time physical activity by 13%, but only partially mediated the effect on walking by 4% at 24 weeks.

**Conclusion:** An mHealth intervention involving text messaging and Internet support had a positive treatment effect on leisure-time physical activity and walking at 24 weeks, and this effect was likely mediated through changes in self-efficacy. Future trials should examine other potential mediators related to this type of intervention.

## Introduction

The ubiquitous use of mobile phones offers important new opportunities to bring self-management support directly to people with long-term conditions such as cardiovascular disease (CVD) (1). Mobile health (mHealth) programs offer several advantages for supporting patient self-management, compared with traditional office-based approaches: (1) they can be delivered anywhere at any time and for extended periods, facilitating regular communication and behavioral maintenance; (2) they can be designed to send messages in a time-sensitive manner that fits with the individual’s lifestyle; (3) they are proactive and do not require prompting by the user before support is offered; (4) they can be personalized and tailored to suit specific demographic and health needs; (5) they increase access (e.g., less travel); (6) they allow cheaper provision of services than face-to-face contacts; and (7) they provide a way of reducing inequalities due to their widespread adoption by all cultural and socioeconomic groups.

Research on the use of mHealth for delivering healthcare and improving disease self-management (2) has increased in recent years (3). This research has targeted a wide range of health conditions (4) and a number of systematic reviews support the delivery of mobile phone text messaging interventions (3–5) for achieving behavior change. However, a recent (2014) meta-review highlighted that the quality of future studies needs to be improved and interventions should employ behavior change theory (4).

A recent randomized controlled trial of a mobile phone text messaging and Internet intervention (HEART) showed a statistically significant treatment effect on self-reported leisure-time physical activity and walking (secondary outcomes) but not on peak oxygen uptake (PVO_2_; primary outcome), which favored the intervention group at 24 weeks (6). In response to the lack of theoretical basis of many mHealth interventions to-date (4) the HEART intervention content development was grounded in self-efficacy theory (7–10), which is a key psychosocial determinant of exercise and physical activity behavior (11–13).

Self-efficacy refers to an individual’s beliefs in his/her capabilities to execute necessary courses of action to satisfy situational demands (9). Self-efficacy is theorized to influence the activities that individuals choose to approach, the effort expended on such activities, and the degree of persistence demonstrated in the face of adverse stimuli (9, 14). Within the cardiac setting, self-efficacy has been the most examined psychological variable and has consistently been shown to be related to exercise behavior (15), exercise intentions (12, 16), and treadmill test performance (13, 17). Furthermore, self-efficacy based interventions have been shown to have a positive effect on exercise behavior (18, 19), adherence to exercise regimens (19–21), and effort expended during bouts of exercise testing (13). There is however, a lack of empirical evidence on the impact of mHealth interventions on efficacious beliefs and the potential mediating effect of self-efficacy on exercise behavior. Understanding potential mediators of mHealth interventions is needed to increase their effectiveness (22). In this paper, the mediating effects of self-efficacy on secondary outcomes of the HEART trial were examined. Mediators identify possible mechanisms through which a treatment might achieve its effects, and represent the causal links between treatment and outcome (23).

## Materials and Methods

A two-arm, parallel, randomized controlled trial was conducted in Auckland, New Zealand between 2010 and 2012. One hundred seventy-one adult participants, with a diagnosis of ischemic heart disease (IHD) were randomly assigned to either receive the HEART mHealth intervention in addition to usual cardiac services (*n* = 85), or to usual cardiac services alone (control group; *n* = 86).

Full details of recruitment, participant flow through the study, and measures have been published elsewhere (24). In brief, participants were recruited from two metropolitan hospitals, and were identified by cardiac nurses as inpatients, through outpatient clinics and existing databases. After screening for eligibility, nurses referred interested participants to the research team to schedule baseline data collection procedures.

Eligible participants were adults aged >18 years, with a diagnosis of IHD (defined as angina, myocardial infarction, revascularization, including angioplasty, stent, or coronary artery bypass graft) within the previous 3–24 months. All participants were clinically stable as outpatients, able to perform exercise, able to understand and write English, and had access to the Internet (e.g., at home, work, library, or through friends or relatives). All participants owned a mobile phone. Participants were excluded if they had been admitted to hospital with heart disease within the previous 6 weeks; had terminal cancer, or had significant exercise limitations other than IHD.

All participants were free to participate in any other cardiac services or support that they wished to use. In addition, participants in the intervention group received a personalized, automated package of text messages via their mobile phones aimed at increasing exercise behavior over 24 weeks. The primary goal of the intervention was to have all individuals participate in moderate to vigorous aerobic-based exercise for a minimum of 30 min (preferably more) most days (at least 5) of the week (25). Intervention content was grounded in self-efficacy and consisted of (1) regular exercise prescription, (2) provision of behavior change strategies, and (3) technical support. Additional information was provided via a secure website that participants could log on to, and included role model video vignettes, an opportunity to self-monitor progress, as well as information on various forms of physical activity and exercise, energy expenditure, healthy eating advice, and links to other websites (e.g., local exercise programs and cardiac clubs). Full details of the intervention are described in the protocol (24).

### Measures

All outcomes were measured at baseline and 24 weeks. The primary outcome (PVO_2_) was determined using respiratory gas analysis during a standardized treadmill exercise testing protocol (26). Self-reported physical activity levels were assessed using the international physical activity questionnaire long form (IPAQ-LF) (27). Self-efficacy (task) was assessed using a valid measure on a scale of 0 “no confidence to 100% complete confidence” (28). An example item is, “how confident are you that you can complete 30 min of physical activity at a moderate effort on most days of next week?” Scores were summed with greater values indicating greater efficacy to exercise for longer periods of time and at a greater level of intensity. For barrier efficacy, participants rated their confidence to overcome seven common reasons (e.g., bad weather, lack of time, pain, or discomfort) preventing people from participating in exercise sessions (12). Efficacy strength was calculated by summing the scores and dividing by total number of items.

In accordance with the recommendations of Kraemer et al. (23) for testing mediators of treatment effects in randomized clinical trials, hierarchical regression analyses were conducted on the observed participants’ data with post intervention physical activity (leisure-time and walking) at 24 weeks as the criterion measures. According to Kraemer et al., a mediator measures an event or change occurring during treatment, and must correlate with the treatment choice, hence possibly be a result of treatment, and have either a main or interactive effect on the outcome. Their analytic approach differs conceptually from that of Baron and Kenny (29) in several important ways. According to Kraemer et al. with mediation, demonstration of precedence is required, thus a mediator occurs during treatment. Similarly, demonstration of correlation is required. In the absence of such criteria, they argue that the interpretation of whether a relationship is mediating or moderating is often arbitrary. The analytic model, in contrast to the several linear model proposed by Baron and Kenny, is exactly the same for moderators and mediators. The difference lies in how M (Mediator or Moderator) is defined in terms of time relation to treatment onset and correlation with treatment choice (23).

## Results

All statistical analyses were performed using SAS version 9.3 (SAS Institute Inc., Cary, NC, USA). Statistical tests were two-sided at 5% significance level. Treatment evaluations were performed on the principle of intention to treat (ITT), using observed data collected from all randomized participants. Analysis of covariance (ANCOVA) regression model was used to evaluate the main treatment effect on the outcome measured at 24 weeks, adjusting for the baseline outcome, age, sex, ethnicity (Māori vs. non-Māori), and exercise history. Change in self-efficacy at 24 weeks was added as the mediator of interest to the main model for evaluation of its mediation effect.

Significant main treatment effects were observed in favor of the intervention for leisure-time physical activity [group difference: 426 MET-min/week, 95% confidence interval (CI) (16, 836), *P* = 0.04] and walking [group difference: 500 MET-min/week, 95% CI (91, 908), *P* = 0.01] at 24 weeks (see Table 1).

**Table 1:** **Treatment effects^a^ with and without self-efficacy as the mediator**.

Outcome at 24 weeks	Intervention	Control	Difference in groups	Lower 95% CI	Upper 95% CI	*P*-value
**LEISURE-TIME PA**						
Main model	1394	968	426	16	836	0.042
Model with mediator^b^	1363	994	369	−37	775	0.075
**WALKING**						
Main model	1690	1191	500	91	908	0.017
Model with mediator^b^	1681	1199	481	68	894	0.023

*^a^Linear regression model adjusting for: baseline outcome, age, sex, Māori, and exercise history*.

*^b^Mediator: change in self-efficacy at 24 weeks*.

Change in task self-efficacy significantly mediated the treatment effect on leisure-time physical activity (*P* = 0.021) by 13% [group difference: 369 MET-min/week, 95% CI (−37, 775), *P* = 0.07], but not on walking (*P* = 0.51) with only 4% relative reduction in treatment effect [group difference: 481 MET-min/week, 95% CI (68, 894), *P* = 0.02]. Barrier efficacy did not meet the conditions for mediation and was not included in the analysis (see Table 1).

## Discussion

The main findings from this study can be summarized as follows. A theory-based mHealth intervention involving text messaging and Internet support had a positive effect on physical activity levels. The effect was most likely mediated through increased self-efficacy to undertake more physical activity at increasing intensity. Self-efficacy can be targeted in mHealth interventions and has potential to increase physical activity behavior.

In the present study, the HEART intervention had a positive effect on task efficacy but not for barrier efficacy. Task efficacy referred to participants’ confidence to exercise for greater intensity and increasing duration. Intervention content (messages and Internet) targeted all sources of self-efficacy, including mastery experiences, social modeling, social persuasion, and physiological responses, which may have had a stronger impact on participants’ confidence to perform exercise, but not for overcoming barriers to exercise. The lack of effect on barrier efficacy was surprising given that the text messages and role models vignettes did address this construct. While items were drawn from a previous study of New Zealand patients with CVD; (12) it is possible that the barriers (e.g., weather, discomfort/pain, work commitments) assessed in this study were not salient for this population. Future studies might need to consider identifying more relevant barriers (30).

Notwithstanding the issues above, this study does provide some support for the intervention successfully manipulating key constructs of Social Cognitive Theory. This is important as it is unclear whether existing behavior change theories are relevant for mobile delivered interventions (31). While mHealth behavior intervention development could benefit from greater application of health behavior theories, Riley et al. (31) argued that current theories appear inadequate to inform such mHealth intervention development as these interventions become more interactive and adaptive. They suggested that consideration be given to the development of more dynamic feedback system theories of health behavior, utilizing longitudinal data from mobile devices and control systems engineering models.

This study has several strengths and limitations that should be considered when interpreting the findings. Data were obtained from an adequately powered RCT, which used computer randomization to ensure allocation concealment, and an objective measure for the primary outcome, with blinded assessors. The HEART intervention was also developed using established theory. The main limitation was the use of a self-reported measure of physical activity behavior, which is associated with recall bias. Objective assessment of physical activity (e.g., accelerometry) was not feasible in this study due to logistic reasons; but should be used in subsequent research.

Opportunities for future research exist. First, the use of alternative theoretical frameworks or embedding key behavior change strategies common to many behavior change theories may enhance the impact of future mHealth interventions (31). Second, other potential mediators (including attitudes to or preferences for physical activity) and moderators (age, sex, ethnicity) of mHealth interventions should be considered. Third and finally, while the HEART intervention utilized text messaging and a website, it was predominantly unidirectional and did not include many interactive features. Future interventions should harness many of the existing native smart phone features including application to enhance participant interaction and encourage behavior change. These could include self-monitoring features such as recording activities and visual representation of progress, passive collection of physical activity data, social media features, as well as opportunity to interact with health practitioners.

## Conclusion

An mHealth intervention had a positive effect on physical activity levels, which was most likely mediated through increases in self-efficacy. Future trials should examine other potential mediators related to this type of intervention, including attitudes to, or preferences for physical activity.

## Author Contributions

Ralph Maddison provided study oversight. Leila Pfaeffli, Karen Carter, and Jonathan Rawstorn conducted the research and undertook data collection. Yannan Jiang performed the statistical analyses. Ralph Maddison, Robyn Whittaker, Ralph Stewart, and Andrew Kerr designed the research (project conception and development of overall research plan). Ralph Maddison wrote the paper. All authors assisted in interpretation of the analyses and revision of the paper. All authors read and approved the final manuscript. Ralph Maddison takes primary responsibility for final content. Ralph Maddison has had full access to all of the data in the study and takes responsibility for the integrity of the data and the accuracy of the data analysis.

## Conflict of Interest Statement

The authors declare that the research was conducted in the absence of any commercial or financial relationships that could be construed as a potential conflict of interest.

## Abbreviations

PVO_2_, peak oxygen uptake.
